# Innovative Polymer Composites with Natural Fillers Produced by Additive Manufacturing (3D Printing)—A Literature Review

**DOI:** 10.3390/polym15173534

**Published:** 2023-08-24

**Authors:** Beata Anwajler, Ewa Zdybel, Ewa Tomaszewska-Ciosk

**Affiliations:** 1 Faculty of Mechanical and Power Engineering, Wroclaw University of Science and Technology, 27 Wybrzeze Wyspianskiego Street, 50-370 Wroclaw, Poland; 2 Department of Food Storage and Technology, Wroclaw University of Environmental and Life Sciences, 25 Norwida Street, 50-375 Wroclaw, Poland; ewa.zdybel@upwr.edu.pl (E.Z.); ewa.tomaszewska-ciosk@upwr.edu.pl (E.T.-C.)

**Keywords:** 3D printing, natural fillers, biofibres, biocomposites, biomass, TPS

## Abstract

In recent years, plastics recycling has become one of the leading environmental and waste management issues. Along with the main advantage of plastics, which is undoubtedly their long life, the problem of managing their waste has arisen. Recycling is recognised as the preferred option for waste management, with the aim of reusing them to create new products using 3D printing. Additive manufacturing (AM) is an emerging and evolving rapid tooling technology. With 3D printing, it is possible to achieve lightweight structures with high dimensional accuracy and reduce manufacturing costs for non-standard geometries. Currently, 3D printing research is moving towards the production of materials not only of pure polymers but also their composites. Bioplastics, especially those that are biodegradable and compostable, have emerged as an alternative for human development. This article provides a brief overview of the possibilities of using thermoplastic waste materials through the application of 3D printing, creating innovative materials from recycled and naturally derived materials, i.e., biomass (natural reinforcing fibres) in 3D printing. The materials produced from them are ecological, widely available and cost-effective. Research activities related to the production of bio-based materials have gradually increased over the last two decades, with the aim of reducing environmental problems. This article summarises the efforts made by researchers to discover new innovative materials for 3D printing.

## 1. Introduction

**Figure 1:**
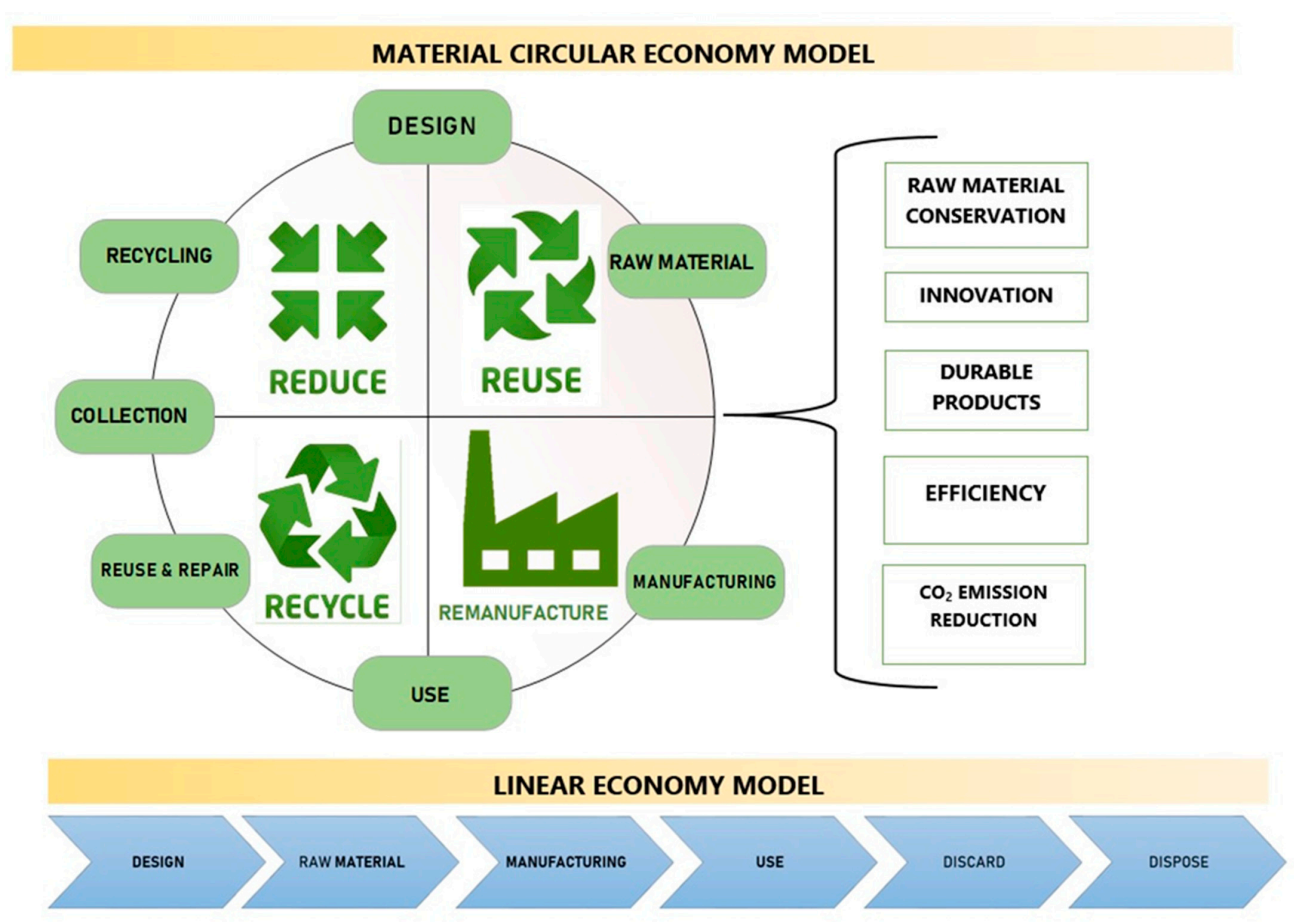
The concept of a circular economy (CE) in comparison with linear economy model. Developed based on [2].

The concept of a circular economy (CE) is a response to environmental and social problems, replacing the linear concept based on the ‘take-produce-throw away’ model. The intensive use of resources and the uncontrolled pollution of the environment have made it necessary to implement a different economic cycle based on the 3R principles: Reduce, Reuse, Recycle. The broader methodology (6R) (Figure 1) includes three additional approaches: Recover, Redesign and Remanufacture [1].

**Figure 2:**
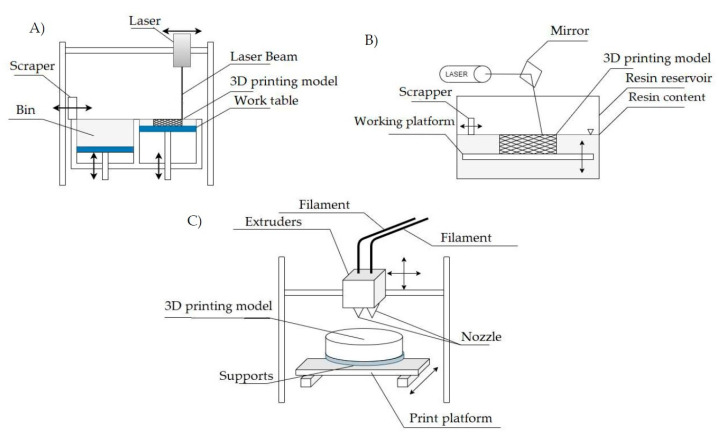
Schematic of the construction of printers using 3D printing technology: SLS (**A**), SLA (**B**) and FDM (**C**).

Additive manufacturing, or (3D) printing, is a transformative manufacturing process that allows the creation of a three-dimensional object using various processes and raw materials such as filaments, resins and powder grains, generally building the product layer by layer [3,4]. There are several types of 3D printing processes, such as FDM—Fused Deposition Modelling, SLS—Selective Laser Sintering, SLA—Stereolithography and many others [5]. Stereolithography (SLA) has been characterised as an example of a technology that uses a raw material in liquid form, and the process of Fused Deposition Modelling (FDM) of thermoplastics has been chosen as an example of a technology that uses a solid building material, and powder laser sintering (SLS) has been discussed as an example of a technology that uses powders. Each of the listed 3D printing techniques has different capabilities depending on its application. The different AM processes are shown in Figure 2.

Fused Deposition Modelling (FDM) [6] uses layered deposition of thermoplastic polymers to print the model. This technique is also known as FFF—Fused Filament Fabrication. The material is fed to the head of the machine in the form of a filament wound on a spool. The filament, as the material is called, is fed through the feed mechanism into the heated nozzle. Under the influence of high temperature, the polymer becomes plastic and is extruded in semi-liquid form. The reciprocal movements of the head and print platform allow the structure to be built up layer by layer. FDM printers are often equipped with a separate nozzle that supplies building material for the creation of supports. Using a separate material for their implementation allows the supports to be easily removed by dipping the print in a suitable solvent. At the same time, the base material must be resistant to this solvent. The main advantage of filament printers is their low cost. This is due to the simple construction of the unit, which also influences the low failure rate and ease of transport. The absence of dust and low noise levels make them suitable for use in offices. The technology makes it possible to reduce the consumption of filling material by using an open work filling of the inside of the model, while maintaining a satisfactory mechanical strength of the pattern. A valued advantage of FDM technology is the ability to build a model in parts and join them together with special glues or grooves. One factor that can deter users from using filament printing technology is the problem of removing the support material from hard-to-reach areas, such as cavities or holes. However, the number of supports can often be reduced by rotating the pattern relative to the plane of the worktop, which is worth considering when importing the model into the 3D CAM software re-sponsible for generating the code for the printing device. The quality of the print walls, particularly those that are inclined relative to the plane of the table top, leaves much to be desired. Visible ‘staging’ is a direct result of the way the FDM machine works, but can be limited by reducing the thickness of the print layer.

Stereolithography (SLA) [6] is considered a precursor to rapid prototyping methods. It is based on curing photosensitive resins with a beam of UV radiation. The laser, which operates in the UV radiation band, directs the beam by manipulating the mirror onto the working platform, which is at the level of the resin mirror at the start of the process. The UV radiation cures the resin layer, then the platform is lowered by the thickness of the layer. The scraper applies some fresh, uncured material to the top of the printed pattern, as fresh resin may not completely cover the model due to its viscosity. This process is repeated until the pattern is complete. The stereolithography process is often used where high printing accuracy is required. The capabilities of the SLA technique allow the maintenance of high resolution compared to other 3D printing techniques. The “stepping” effect on sloping model surfaces is limited. The SLA process can also be used to produce thin-walled patterns and models with a high degree of geometric complexity. Resin printing is popular in applications where transparency of the prototype is important. The disadvantage of SLA printers is the need to clean samples from liquid resin residue and drying. The toxicity of the liquid resin prior to polymerisation is also a concern. Stereolithography prototypes can lose their properties at temperatures above 40 °C. Unlike some technologies, SLA printers cannot produce a model from multiple materials in one process.

Selective Laser Sintering (SLS) technology [6] is based on the laser sintering of powders. The most commonly used powders in this technology are plastic powders, which are characterised by favourable properties of the printed model and susceptibility to sintering. The resulting sinter is not a uniform material—it is porous in nature. To increase the density and strength of the model, the structure is often infiltrated with another substance. Plastics are not the only materials that can be sintered. Metal powders can also be sintered. Plastic powder sintering machines build a model by applying thin layers of material and then burning the appropriate path with a laser. The powder is placed in a hopper, the capacity of which is adapted to the requirements of the item being produced. The scraper applies thin layers of powder to the work surface, which is reduced by a predetermined cross-sectional thickness after each laser operation. Once the finished product has been removed from the hopper, it must be cleaned to remove any remaining loose material. The most commonly used method is glass blasting of the sample surface. The undeniable advantage of the SLS method is the ability to print objects without the need of any supports—unsintered powder acts as a support. This feature is a great advantage when printing structures with a complicated internal structure where the traditional support structure cannot be removed. The wide range of materials available means that the technology can be used to print models with different properties, including flexible objects. Unfortunately, SLS printing also has some drawbacks. Selective laser sintering prevents the printing of empty, closed volumes. These spaces are filled with powder, as it is impossible to get rid of the filling without mechanically damaging the model. For this reason, SLS is the preferred method for printing open-cell structures. The problem of cleaning the object from the remains of loose building material can also cause a lot of problems. The printer’s workspace is heated, which tends to sinter powders with a low softening point. While simple structures do not cause problems during the cleaning process, the complex structure of the object, with its many small depressions and high porosity, favours complications during post-print processing. Due to the nature of the printing process, the SLS method does not allow the use of different types of construction materials without mixing them within a sample.

Additive manufacturing (AM) technology has the advantages of reducing raw material consumption, reducing energy consumption during production, reducing cost, time, etc. It is flexible in terms of product manufacture and is not limited by the shape and structure of the product [7]. Incremental methods have been particularly successful in medicine [8,9] and various fields such as engineering [10], agri-food [11,12] or pharmaceuticals [13]. Fast production times and a simple computer-aided design (CAD) process are key to the growing success of incremental manufacturing technology [14]. 3D printing technology has the potential to produce functional products from a wide range of polymers and polymer composites [14,15]. Fused Deposition Modelling (FDM) is the most popular AM technology due to its rapid production, cost-effectiveness, ease of access, wide material adaptability and ability to produce complex components [15]. 

The polymers used in 3D printing can be divided into three groups according to their origin: synthetic, semi-synthetic and naturally biodegradable [16]. Synthetic polymers are made from petroleum and are not biodegradable. Semi-synthetic polymers are intended to be biodegradable, and naturally biodegradable polymers are derived from natural resources. Examples of the former are PVA—polyvinyl alcohol, aliphatic and aromatic polyesters, PBAT—polybutylene adipate terephthalate, PBS, PCL—polycaprolactone, PTT—tri-methylene terephthalate, PA—polyamide, ABS—acrylonitrile butadiene styrene, PE—polyethylene, PP—polypropylene, PC—polycarbonates, PEI—polyethyleneimine, PEKK—polyetherketoneketone, PS—polystyrene and PEEK—polyetheretherketone. The second type is microbial polymers (e.g., polyhydroxyalkanoate (PHA)) and synthetic polymers from monomers (e.g., PLA, polyglycolic acid (PGA) and polybutylene succinate (PBS)), while the third type includes starch, chitosan, cellulose, hemicellulose, lignins and protein [16].

**Figure 3:**
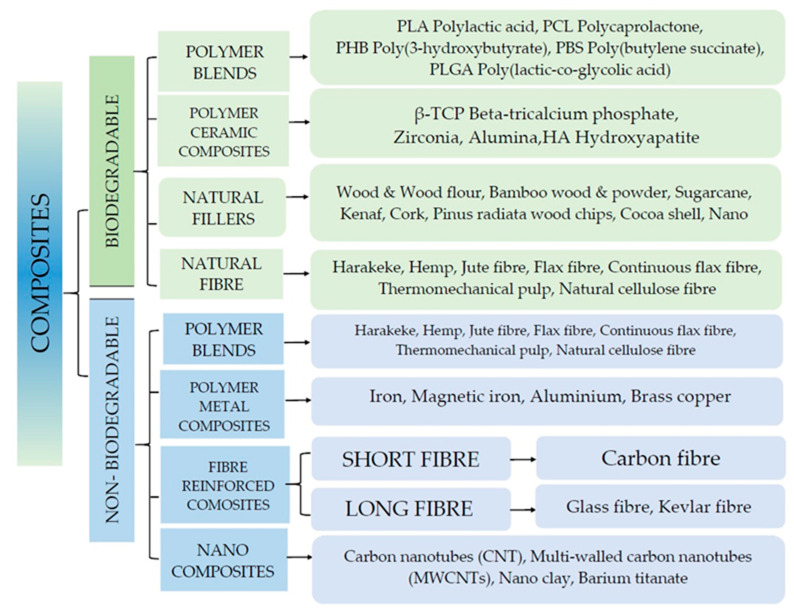
Materials in additive technology. Developed based on [15].

Currently, non-biodegradable materials are mainly used for 3D printing. To overcome these limitations, research has begun to explore the possibility of using different types of bio-derived materials [16]. Therefore, the production of composite materials compatible with current printers has gained considerable interest in recent years. In the available literature, many promising discoveries have been reported for the production of new composites for printing, reinforced with ceramics, metals, fibres and nanomaterials [15]. The materials used with polymeric materials are mainly divided into two types: (1) biodegradable materials and (2) non-biodegradable materials—Figure 3 [15]. In recent years, with the emphasis on green and low carbon concepts, research on biomass materials in 3D printing has been progressively developed and extensive review articles have been published in this field [7]. The article reviews the progress made in recent years in the research of various biomass 3D printing technologies and defines the prospects for the development of 3D printing with the addition of natural fibres.

## 2. Biodegradable Materials and Polymer Composites with Natural Fillers

Biodegradable materials are natural materials and their properties are relatively inferior to those of non-biodegradable materials [15,16]. Biofibre reinforcement or blending with other biodegradable products has proven to be an effective way to reduce the cost and brittleness of some commonly used polymers to produce a fully biodegradable composite. PLA and ABS polymers are standard materials used as base materials in composites due to their low cost, easy availability and good mechanical properties [15,16]. Inorganic or organic materials such as glass, carbon fibres, silicon, ceramics or metals have been used in research to date. A more recent option is the use of natural polymers (polysaccharides and proteins) as reinforcing agents, derived from plants or biomass, possibly from agricultural or industrial waste. The main advantage of natural materials and biofibres as fillers for 3D printing is their availability. Composites made with them have less negative environmental impact and are more biodegradable. They are therefore considered a ‘green option’ for 3D printing processing [4,5,16,17]. Further development, production and testing of composites for 3D printing is therefore required to improve mechanical and performance properties. Therefore, the use of natural materials as reinforcing agents for 3D printing is a new strategy that offers an attractive alternative to all fillers of synthetic and non-renewable origin [4]. 

Polymers are often reinforced with fillers to improve their thermomechanical properties. Especially when the fillers are derived from natural resources, they help to achieve optimal properties such as biodegradability and biocompatibility. In addition, increasing urbanisation has led to a huge increase in agricultural, industrial and household waste. The synthesis of bio-based fillers from these resources at the nanoscale enables the hybridisation of bio-based fillers with synthetic and natural polymers [4,5]. Many natural plant fillers are extracted from agricultural by-products and consist of a mixture of different biopolymers such as cellulose, lignin and hemicellulose, which are components of plant parts (e.g., leaves, seeds, fruits, grasses) [4]. Biofibres are generally considered organic and sustainable due to their versatility, eco-friendly design, low cost, renewability and local supply compared to synthetic fibres [18]. As in nature, these fibres are hollow and have very good thermal and acoustic insulation properties. The mechanical properties of biofibres are generally lower than those of synthetic fibres, but careful surface preparation can improve these properties [4,18]. However, the main disadvantages of biofibres are their hygroscopicity and incompatibility with the hydrophobic matrix [4,16,17].

Researchers involved in the development of a natural fibre for FDM are listed in Table 1 [19]. Typical natural fibres used for biocomposite production are oil palm, banana, kenaf, walnut shell, rice, rice straw, wood, Hedysarum, agave, sugarcane, astragalus, bamboo, almond hull powder, etc. The inclusion of processing makes the print accurate.

Biomass polysaccharides are macromolecules composed of repeating sugar units linked by glycosidic bonds to form a crystalline and amorphous material. They are abundant in nature and many have a complex structure consisting of numerous intramolecular and intermolecular hydrogen bonds. The most representative components of this class are cellulose, hemicellulose, lignin, chitosan, starch and alginate [9]. Polysaccharides have been used as reinforcing agents in the form of pure fibres (e.g., cellulose, lignin or hemicellulose) or as mixtures extracted from plants (e.g., flax, bamboo, hemp) of which they are the main constituents. Other sources of polysaccharides are process waste from plant matrices, such as coffee. Proteins [76] are complex macromolecules made up of amino acids linked by peptide bonds. They are abundant in nature and have beneficial properties such as biodegradability and biocompatibility. However, they are mainly used in the 3D process to build soft materials such as hydrogels or scaffolds for tissue engineering. Proteins used for 3D printing include gelatin, keratin, collagen, silk and soy proteins [77].

### 2.1. FDM 3D Printing with Natural Fibers

The most common of the various 3D printing techniques is FDM, and its success depends on the availability of materials that can be processed with it [8]. Different types of fibres are available, including biological (natural) fibres.

**Figure 4:**
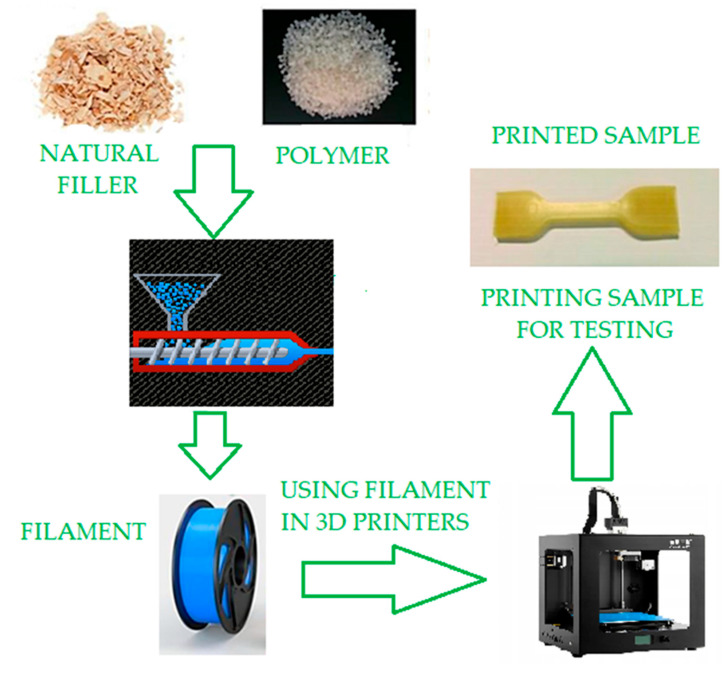
Schematic of 3D printing of bio-based polymer composites. Developed based on [19].

Natural fibres are a very popular choice due to their low cost, abundant availability, high strength-to-weight ratio and high aspect ratio and modulus of good strength and elasticity. These properties make biocomposite filaments a good substitute for synthetic fibres [17]. The general process for obtaining biocomposite polymer parts from FDM is shown in Figure 4. They can also be obtained from mixed biomass and petroleum sources. Commonly used biopolymers are PHA, PEG, PCL and PLA [19].

PLA is one of the most widely used biodegradable plastics for FDM. Synthesised from agricultural resources such as corn and tapioca, PLA is biocompatible, compostable, recyclable, gas permeable and degradable by hydrolysis and enzymatic action. PLA is well suited to FDM printing due to its low melting point, low coefficient of thermal expansion and lack of odour during processing. However, it is significantly more expensive per unit than petroleum-based plastics such as polyethylene and polypropylene. It also has a longer degradation time. Therefore, there is an urgent need to reduce the cost of PLA and improve its degradability [78]. Biofibre reinforced PLA composites have received much attention in recent years. By mixing a biodegradable matrix with a biofibre reinforcement, it is practically possible to produce a biocomposite, a fully biodegradable material [19]. Work on biofibre-based PLA composites has shown some advantages such as high processability, high specific elasticity, compostability, high durability, renewability and recyclability [19,78].

According to Hu and Lim et al., the mechanical properties of the harakke composite were superior to those of conventional PLA [15,79]. Harakeke was added to PLA at 30, 40 and 50 wt%, and the results indicated that the 40 wt% composite had the highest mechanical properties. Le Duigou et al. [15,80] experimented with a PLA/continuous fax fibre (CFF) composite. PLA/jute fibre and PLA/fax fibre composites were investigated by Hinchclife et al. [15,81]. The size of the jute fibre composite was 2 mm and that of the fax fibre was 0.5 mm. The results showed that the tensile strength increased by 116% and 26%, respectively. The stiffness of the product increased by 12% and 10%, respectively. The effect of different l/d ratios of PLA/bamboo fibre and PLA/flax fibre was investigated by Depuydt et al. [15,34] and an increase in stiffness was found. Le Duigou et al. [15,82] investigated and demonstrated the feasibility of printing a hygromesh biocomposite from a PLA/wood fibre composite with a specific bilayer microstructure. The mechanical properties and potential of polypropylene reinforced hemp and harakke were investigated by Milosevic et al. [15,26]. They reported improvements in tensile strength and Young’s modulus of 50% and 143%, respectively, compared to pure polypropylene. The mechanical properties of thermomechanical pulp (TMP) reinforced with BioPE composite were analysed by Tarrés et al. [15,83], who reported an improvement in printing quality. Thibaut et al. [15,84] investigated the mechanical properties and anisotropic shrinkage of carboxymethyl cellulose (CMC) with natural cellulose fibre during drying. The result showed that 30 wt% of the composite had better mechanical properties and lower shrinkage. Several studies have been carried out to improve and analyse the performance of cellulose fibres as textile composites. Cellulose nanofibrils (CNFs) and nanocrystals (CNCs) have been widely used as a modern collection of nanomaterials in 3D printing [9,15,17]. The incorporation of CNCs into PLA improves mechanical and thermal properties as the cellulose nanoparticles act as nucleating agents for crystallisation. The load is absorbed by the CNC particles, which are oriented in the polymer chains, resulting in improved tensile properties [9,17]. Cellulose nanocrystals, obtained by acid hydrolysis of plum seed shells, were successfully incorporated into the PLA/PHB matrix through a reactive mixing process, resulting in improved adhesion and subsequent thermal stability and mechanical properties. Nanocellulose was obtained from the microcellulose using ultrasound treatment to break the aligned structure to produce fully biodegradable 3D printed nanocomposites based on biopolyesters such as PLA, poly(3-hydroxybutyrate-co-3-hydroxyhexanoate) (PHBH) and nanocellulose. The inclusion of nanocellulose in the fibre samples improved the thermomechanical properties of the composites [9,17].

Filaments for 3D printing with improved mechanical strength have been produced from low-density polyethylene reinforced with nanofibrillated cotton (NFC) particles synthesised from cotton material derived from recycled T-shirts [85]. PLA/lignin biocomposites for 3D printing applications have been developed using lignin synthesised from the cooking liquor of common spruce chips using a soda boiling technique. The PLA/lignin biocomposites prepared in this way showed good printability without agglomeration. Due to the antioxidant effect of lignin, these composites also showed enhanced uptake activity. Carbon nanoparticles synthesised from waste coconut shell powder are incorporated into Bioplast to develop biodegradable fibres for 3D printing [17]. In addition to the nanofillers mentioned above, several other industrial and plant by-products are used as fillers for polymer composites [17].

Carbon fibre from coffee beans acts as a thermal insulator [17]. Ox-SCG and PLA composite filaments can be produced using a single-screw FDM filament extruder. In addition to PLA, Ox-SCG has also been used with other materials. For example, Huang et al. used coffee grounds with a polyethylene matrix, resulting in an overall increase in modulus and thermal properties [17,86], while Moustafa et al. used coffee grounds with polybutylene adipate catheterephthalate. Hung et al. [86] prepared the filaments by mixing powdered PLA with Ox-SCG at concentrations of 0, 5, 10, 15 and 20 wt% with a total weight of 100 g per mixture. The mixture was prepared in a standard V-type powder granulation mixer for 4 h. The mixture was then held in a vacuum oven at 40 °C to remove moisture from the mixture. The resulting mixture was fed into the hopper of a single-screw extruder at 165 °C. The composite was extruded to a diameter of 1.75 mm and then coiled. The resulting filament was suitable for 3D printing. Hung et al. [86] reported that Ox-SCG was uniformly dispersed and distributed in the PLA matrix by mixing and single-screw extrusion. They also reported that increasing the concentration of Ox-SCG in the PLA matrix increased the strength. At 20 wt% Ox-SCG loading, an improvement in impact strength of 418.7% was reported [17].

Wood and lignocellulosic components can also be used as additives and reinforcements in composites [15]. The search for fillers such as wood, bamboo, sugarcane, kenaf with PLA and other base materials is ongoing. Ayrilmis et al. [15,87] investigated PLA with 30 wt% wood using FDM. They investigated the absorption and changes in mechanical properties at different layer thicknesses: 0.05 mm, 0.1 mm, 0.2 mm and 0.3 mm. The results indicate that increasing the layer thickness increases the porosity and decreases the mechanical properties of the sample. PLA/raw sugar cane and PLA/raw sugar cane fibre were investigated at different compositions of 3, 6, 9 and 12 wt% by Liu et al. [88] and found to have the best properties for industrial scale applications. A study on the preparation of bamboo/PLA composites using FDM was carried out by Zhao [15,89]. It was found that the addition of bamboo powder to the PLA polymer reduced nozzle clogging and had better biodegradation properties. Daver et al. [15,90] investigated the morphological, mechanical and thermal properties of PLA filled with cork at different filling percentages. The tensile strength and yield strength of the printed parts were low compared to the compression moulded composites, but the elongation at break was higher. A PLA/wood flour composite was investigated by Tao et al. [35]. Their results indicate that the addition of 5 wt% wood flour to PLA does not change the melting point of the composite. Vaidya et al. [15,91] analysed the warpage of the composite in relation to the addition of polyhydroxybutyrate (PHB) fillers and Pinus radiata wood chips. A 20 wt% filler added to PHB changes the melt viscosity and improves warpage from 34 to 78% compared to parts printed from pure PHB. Tran et al. [92] investigated the thermal and mechanical properties of a polycaprolactone (PCL)/cocoa husk composite. Varying the composition of cocoa husks added to PCL resulted in a low temperature composite suitable for printing biomedical scaffolds and toys. Frone et al. [93] investigated the morphostructural and thermomechanical properties of nanocrystalline cellulose added to a polylactic acid (PLA)/polyhydroxybutyrate (PHB) composite and dicumyl peroxide (DCP) as a crosslinking agent. Good adhesion and thermomechanical properties of the sample were reported.

Thermoplastic starch (TPS) [94], as a cheap, readily available and fully biodegradable and renewable polysaccharide, has found its application as an additive in composites with degraded polymers, i.e., PLA, PBS and PCL. TPS is obtained by subjecting starch to the appropriate moisture, mechanical shear stress and heat. Research [95] has shown that the resulting material is susceptible to ageing and brittleness. In addition, the mechanical properties and hydrophobicity of TBS are poor compared to traditional materials such as polyethylene or polypropylene. This is due to the large number of hydroxyl groups, which contribute to strong intermolecular interactions [78]. Various strategies have been developed over the years to improve these properties, including the use of plasticisers, reinforcement with reactive modifiers and chemical modification of starch and blending starch with other polymers [96]. Combining starch with other polymers in FDM 3D printing has proven to be an effective way to overcome the limitations of thermoplastic starch. According to the available literature, numerous studies have been carried out on the use of TPS as a matrix in FDM printing, and the resulting blends, e.g., TPS/PLA, have proven to be an excellent solution for obtaining new and inexpensive materials with good performance. Other advantages of these composites include renewability, biodegradability, low density and high heat resistance. The only drawback is poor durability, which has become a key element in further research by scientists [96].

**Figure 5:**
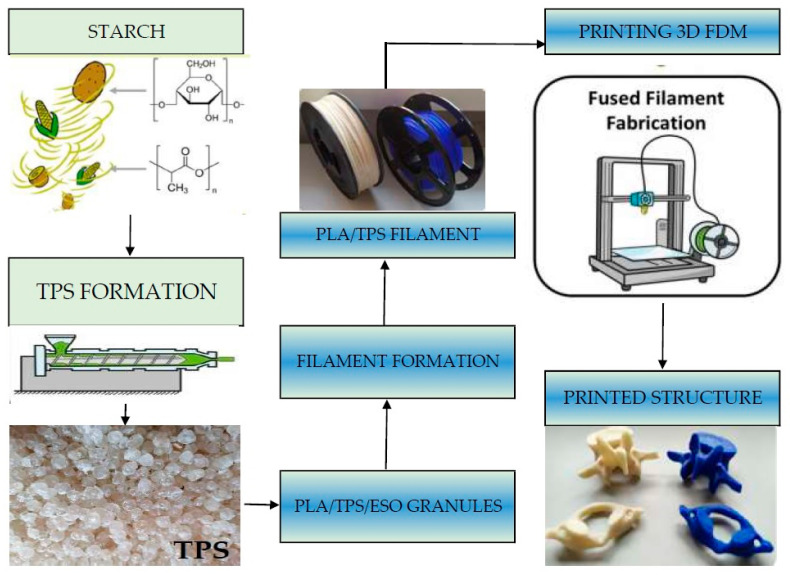
Block diagram showing the steps of PLA/TPS 3D printing. Developed based on [94].

Haryńska [94] started her research with the preparation of a composite based on thermoplastic starch (TPS) and PLA in the proportion of 60 wt% PLA and 40 wt% TPS. The TPS granules were prepared from potato starch, vegetable glycerine and ESO soybean oil in the following proportions 65.7% starch, 33.3% glycerine and 1% ESO. The resulting material was used for FDM 3D printing and then its mechanical properties were tested (Figure 5).

Ultimately, the researchers were able to increase the biodegradability and plasticity of PLA. The improvement in printability was also demonstrated by producing personalised 3D anatomical specimens and prints with complex shapes and high structural porosity. Researchers [95] produced composites based on mTPS/PLA in a ratio of 75 wt.%. PLA and 25 wt% mTPS using the ESO additive in the preparation of TPS in the amount of 0.5–2%. The results obtained showed that the use of 25% thermoplastic starch increased the impact strength of the composites from 13.70 kJ/m^2^ to 16.69 kJ/m^2^ and the elongation at break from 2.6% to 8.8% compared to pure PLA. The authors showed that the use of an additive for the production of TPS with ESO, in the obtained composite with the weight composition TPS/PLA (2% ESO) (25 wt.%/75 wt.%), improved the water resistance and increased the impact strength to over 16 kJ/m^2^. In addition, the thermal and rheological properties, as well as the biodegradability and compostability (according to the PN-EN 14806:2010 standard [97]) of the obtained 3D printed samples were determined. In conclusion, it can be clearly stated that the addition of 25% TPS with ESO to the structure of the PLA composite allows not only to reduce the production costs of the obtained material, but also to maintain the mechanical properties at the level of pure PLA and additionally to improve the biodegradability and compostability of the obtained materials.

Qing Ju [96] prepared three-component blends containing PLA, TPS and PBAT (poly(butylene adipate-co-terephthalate)). In addition, the functional polymer CE (ADR4468) was introduced in different proportions to reduce the brittleness of the obtained composites. The tests were carried out on a composite prepared in the following proportions: 50% TPS, 40% PLA and 10% PBAT. The main component that increases the flexibility of the composites produced is PBAT, which is characterised by high flexibility due to its high molecular weight and branched molecular structure. It also has a density similar to LDPE and is biodegradable. Due to its excellent flexibility, it can be successfully used for rigid two-component TPS/PLA composites. The studies carried out by [96] also found that the mechanical properties improved with increasing chain extender (CE) content, with the addition of CE increasing the elongation at break of the samples by 113% and the impact strength by 190%. However, the maximum viscosity and strength of the samples from the composite obtained were obtained with 1% w/w CE. Furthermore, the addition of CE not only increased the relative molecular weight of PLA and PBAT, but also increased their degree of branching, thereby improving the matrix strength of the four-component composite. At the same time, the applied CE improved the adhesion and compatibility between the two-component blend of PLA and PBAT, which increased the stress and strain efficiency and improved the flexibility of the printed samples.

In the work of Zhao [98], the researchers prepared composites consisting of poly-caprolactone (PCL) and starch in different proportions (from 1 g to 10 g) with the aim of using the material in low temperature FDM printing in a temperature range of 80–90 °C. As a result, it was also possible to introduce PHMB into the matrix of the composite. Polyhexanide (PHMB) is a water-based primer based on acrylic resin dispersion. It reduces and balances the absorbency of the substrate and stabilises and strengthens dusty substrates. It is mainly used as a disinfectant and antiseptic. PHMB has been shown to be effective against *Pseudomonas aeruginosa*, *Staphylococcus aureus*, *Escherichia coli*, *Candida albicans*, *Aspergillus brasiliensis*, *Enterococci* and *Klebsiella pneumoniae*. The use of PHMB contributed to the production of antibacterial and biocompatible materials using FDM 3D printing.

Kuo [99] prepared composites based on ABS (70 wt%) and TPS (30 wt%) with the addition of a compatibiliser in the form of a styrene-maleic anhydride copolymer (SMA); an impact modifier, i.e., methyl methacrylate-butadiene-styrene (MMA); and pigments, i.e., titanium white (TiO_2_) and carbon black (CB). The twin screw extruder produced white and black filaments, all 1.75 mm in diameter. Tests were carried out using the FDM 3D printing method to determine their physical properties. The results of the research, which included flexural modulus, tensile and flexural strength, HDT, MI and impact strength, showed that it was not possible to produce composites with better physical properties than pure ABS. However, the researchers showed that the addition of various amounts of SMA improved the thermal stability, flowability and mechanical properties of the samples obtained, which means that SMA is an effective compatibiliser in the case of a two-component blend of TPS and ABS. In addition, the researchers showed that the addition of CB contributed to better thermal stability, flowability and mechanical properties in the produced ABS/TBS composites compared to the addition of TiO_2_.

Research into the use of starch in FDM 3D printing technology is still giving scientists sleepless nights. They are still trying to expand the use of starch, which is readily available and cheap, and to improve the biodegradability of composites produced by FDM printing.

In summary, there are only a few cases where the addition of natural fibres has led to an improvement in all the mechanical properties of the composites produced. In most cases, a decrease in mechanical properties was observed with increasing biomass filler. The materials used in the FDM process have different ranges of strength and modulus. A wide range of materials have a maximum tensile strength of 40 to 70 MPa and a Young’s modulus range of 0.5 to 2.5 [15]. It is difficult to evaluate each of the composites and biocomposites obtained in this way because they have found applications in different areas of human life. From biomedicine, electronics and construction to furniture, food and textile industries. The technique is still evolving and further research is needed to realise the potential of readily available and inexpensive natural fibres in FDM printing.

### 2.2. SLS 3D Printing with Natural Fibres

The most commonly used materials for SLS 3D printing are nylon, polycaprolactone, sand, metal and wax, which are non-renewable resources. According to the literature, there is a need to find low cost, environmentally friendly and biocompatible materials that could be successfully used with common polymers in SLS 3D printing [7,100].

The authors Guo et al. [101] successfully developed a plastic composite powder (RPC) from rice husk and plastic hot melt adhesive (PMA). Rice husk is a green and biological material and its main advantage is its low cost. The authors developed a special method to mix the substrates and finally obtained a composite that met the requirements of SLS processing in terms of mechanical properties and dimensional accuracy of the prints.

Li et al. [102] prepared composites suitable for SLS by mixing pine wood powder with phenolic resin. The content of pine powder in the mixture varied from 30 to 50 wt%. The authors attempted to find a correlation between pine meal content and electrical conductivity and mechanical properties. The physical properties, microstructure, pore distribution and electrical conductivity of the printed porous carbon electrodes were investigated by scanning electron microscopy, X-ray diffraction and electrical conductivity tests. The results showed that pine wood composites can be fabricated into highly tailored carbon electrodes with high porosity using the SLS 3D printing process. In addition, it can be reported in the literature that Zeng et al. [103] investigated the effect of laser intensity on the mechanical properties of wood plastic composite (WPC) mouldings. As the laser intensity increased from 226 W/mm^2^ to 311 W/mm^2^, the tensile strength improved by 191% and the flexural strength by 17%. Similarly, as the laser intensity increased from 226 W/mm^2^ to 340 W/mm^2^, the impact strength of the raw parts improved by 543% and the impact strength of the wax-impregnated parts improved by 147%. However, the authors also showed that at laser intensities above 311 W/mm^2^, tensile and flexural strength decreased.

Zhang et al. [7,104,105] aimed to address the low strength and low relative density of carbon nanotubes (CNTs) and wood-plastic composites. Microwave processing was used to post-treat the SLS moulded parts. Results showed that microwave treatment for about 60 s could improve the flexural strength of CNT/WPC by 4.2–64.2%. Adding a small amount of Al powder as a reinforcing material in WPC to accelerate heat transfer can solve the problem of low mechanical properties of WPC. Keratin-based materials have unique biological and mechanical properties due to their molecular structure and protein organisation. It is extracted from a variety of common natural sources (wool, chicken feathers and horns) and has numerous beneficial properties including biocompatibility, biodegradability and mechanical strength [16]. Singamneni et al. [106] prepared keratin composites from polyamide and polyethylene for SLS processing. Mechanical characterisation of the samples sintered at the best laser energy densities showed that the presence of keratin significantly weakens the intermolecular bonding achieved by sintering for the keratin-PA combination, while there is a marginal loss for the keratin-PE composite. The results showed no significant improvement in the mechanical properties of the composites.

### 2.3. SLA 3D Printing with Natural Fibres

The raw materials used for SLA are mainly derived from petroleum-based substrates [7]. In order to meet the change in incremental SLA production towards sustainability, the study of renewable raw materials has become as important a research element as for other 3D printing methods. Unfortunately, there are not many options that can be used for photopolymerisation. However, some vegetable oils can be modified to be suitable [107,108] Vegetable oils are renewable raw materials with high performance, biodegradability, low toxicity and surface modifiability that are very promising and applicable to SLA [7]. The authors Anda et al. [107] investigated acrylated epoxidised soybean oil (AESO) and blends of AESO with vanillin dimethacrylate (VDM) and vanillin diacrylate (VDA) as photosensitive resins for optical 3D printing without photoinitiator and solvent. With a higher yield of the insoluble fraction, they obtained better thermal and mechanical properties of the pure AESO polymer. They subjected the plant-derived resins to ultrashort pulse laser polymerisation by multiphoton absorption and avalanche-induced crosslinking without photoinitiator. They confirmed for the first time that pure AESO and blends of AESO and VDM can be used for 3D microstructuring using direct laser lithography.

**Figure 6:**
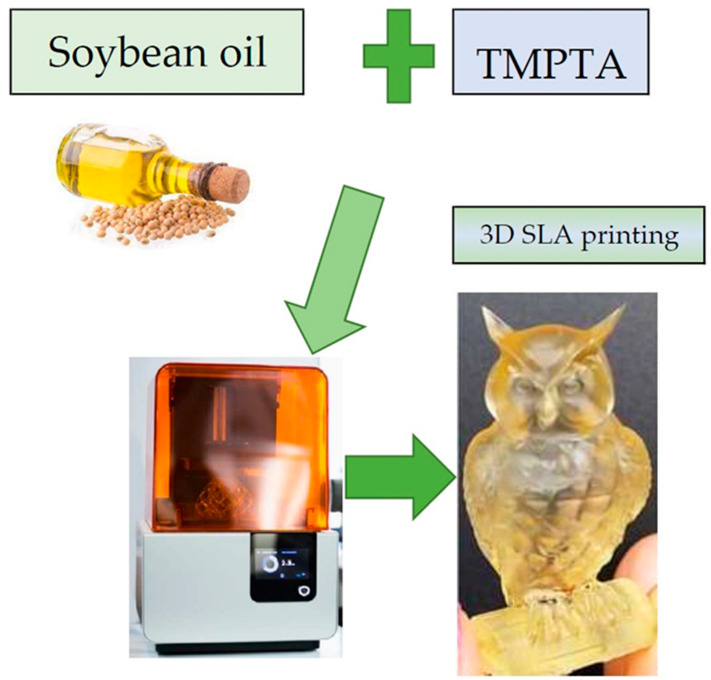
Printed M-AESO-3. Developed based on [108].

In [107], the authors carried out an extended study using FTIR measurements and photoreology on the UV curing of epoxidised acrylates from soybean oil-based formulations (AESO). The study showed that by adding appropriate functional comonomers, such as trimethylolpropane triacrylate (TMPTA), and adjusting the photoinitiator concentration in the range of 1% to 7%, the UV exposure time could be reduced by up to 25%. Thermal and mechanical properties were also investigated using TGA and DMA measurements, which showed significant improvements in mechanical parameters for all formulations. The properties were further improved when reactive diluents were added. After thorough testing, the prepared vegetable oil-based resin ink formulations with reactive diluents were found to be suitable inks for UV-assisted AM, giving them the appropriate viscosity (Figure 6).

Tour’s team and Lin’s team [109] jointly synthesised inks with light-curing, biodegradable and renewable properties using soybean oil, natural polyphenols and luminescent graphene. They also successfully recycled inks from printed products and upgraded biocomposites into luminescent graphene.

## 3. Conclusions

In summary, natural fibre composites are and will be particularly important for the development of a green and sustainable plastics economy. As mentioned in the introduction, this is mainly due to the fact that natural fibres are agricultural and food industry by-products with large reserves, inexhaustible resources, cheap and fully biodegradable. However, one factor that may limit the widespread use of this raw material is the skilful preparation of fibres for 3D printing in order to obtain the best physical properties of the resulting composites.

In the context of the green and low carbon era, the efficient use of biomass and biofibre materials is one of the important directions to promote environmentally sustainable development. The use of 3D printing as an advanced production technology with low energy consumption, high efficiency and easy personalisation is particularly important. Therefore, it is believed that the combination of abundant biomass feedstock and advanced 3D printing technology will provide an environmentally friendly, low-carbon and efficient way for the sustainable development of the materials manufacturing industry. With the development of bioplastics, it is necessary to reduce the consumption and increase the reuse and recycling of plastics. These actions are in line with what the circular economy preaches.

There is still a definite need for future research into polymer-based biocomposites in order to successfully modernise the importance of 3D printing technology. For this reason, the research and development of biopolymers is of great interest to materials science and technology research, both from a scientific and environmental point of view. The authors of this article have also addressed the issue of fabrication and testing of composites based on polyamides and thermoplastic plant starch (TPS) using SLS 3D printing technology. The results of their work will soon be the subject of their next publication.

## Figures and Tables

**Table 1:** Summary of biomass materials used for 3D printing.

Printing Method	Biomass Materials	References
FFF/FDM DIW, LOW, LDM	Composites of oil palm fibre with ABS	Ahmad [20]
	Composites of banana fiber with plastic	Singh [21]
	Composites of kenaf with plastic	Han [22]
	Composites of macadamia nut shells with plastic	Girdis [23]
	Composites of rice straw with ABS	Osman [24]
	Composites of wood dust fibre with plastic	Nafis [25]
	Composites of hemp or harakeke fibres with plastic	Milosevic [26]
	Composite of Hedysarum coronarium flour with PLA	Scaffaro [27]
	Composites of agave fibres with PLA	Figueroa [28]
	Composites of kenaf with PLA	Shahar [29]
	Composites of kenaf with PLA	Jamadi [30]
	Composites of kenaf cellulose with PLA	Liu [31]
	Compound of Astragalus with plastic	Yu [32]
	Composites of natural rubber with PLA	Fekete [33]
	Composites of bamboo and flax fibre with PLA	Depuydt [34]
	Composites of wood flour filled with PLA	Tao [35]
	Composites of soy hulls and soy protein with PLA	Dey [36]
	Lignin powder	Dominguez-Robles et al. [37]
	Lignin sulfonate	Mimini et al. [38]
	Lignin hydrogel	Bonifacio et al. [39]
	Hemicellulose composite	Shi et al. [40]
	Bamboo compounded with plastic	Long et al. [41]
	Compound of wood with plastic	Kariz et al. [42]
	Compound of straw with plastic	Yu et al. [43]
	Compound of cellulose	Ambone et al. [44]
	Lignin with plastic	Ryu et al. [45]
	Wood plastic wire	Yang et al. [46]
	Wood plastic composite	Liu et al. [47], Rahim et al. [48], Tascioglu et al. [49], Fico et al. [50], Cano-Vicent et al. [51], Baechle-Clayton et al. [52]
	Bamboo wood	Muller et al. [53]
	Straw	Yu et al. [54]
	Wheat	Zheng et al. [55]
	Rice	Liu et al. [56]
	Corn	Paggi et al. [57]
	Sugar cane	Nida et al. [58]
	Nanocellulose	Latif et al. [59]
	Cellulose nanofiber	Shin et al. [60]
	Cellulose nanocrystals	Vorobiov et al. [61]
	Cellulose acetate	Huang et al. [62]
	Nano-fibrillated cellulose	Tuladhar et al. [63]
	Microcrystalline cellulose	Murphy et al. [64]
	Hemicellulose paste	Bahcegul et al. [65]
	Galactoglucomannan	Xu et al. [66]
	Hemicellulose hydrogel	Shi et al. [40]
	Paper	Travitzky et al. [67]
	Wood veneer	Liu et al. [68]
	Cellulose powder	Bouzidi et al. [69]
	Wood chips	Rosenthal et al. [70]
	Cellulose ionic liquid	Markstedt et al. [71]
	Cellulose hydrogel	Hu et al. [72]
SLA	Epoxy acrylate soybean oil (AESO)	Rosa et al. [73]
	Lignin-based photosensitive resins	Sutton et al. [74]
SLS	Wood plastic pellets	Zhang et al. [75]
